# Supplementary material for: Polyketide synthase gene clusters in the ericoid mycorrhizal fungus Oidiodendron maius: genome-wide prediction, expression profiling, and functional characterization of knockout mutants
Source: Front Plant Sci. 2026 Sep 16;17:1924430. doi: 10.3389/fpls.2026.1924430 (PMC13623903; doi:10.3389/fpls.2026.1924430)
Supplement: Supplementary file 1 [file SupplementaryFile1.zip › Supplementary Materials/Supplementary Folder 1.zip!/Oidma1_AssemblyScaffolds_Repeatmasked/knownclusterblast/cluster53/jgi.p_Oidma1_173318_mibig_hits.html]

| MIBiG Protein | Description | MIBiG Cluster | MiBiG Product | % ID | % Coverage | BLAST Score | E-value |
| --- | --- | --- | --- | --- | --- | --- | --- |
| AAG27131.1 | Fum3p | BGC0000062 | Fumonisin\_biosynthetic\_gene\_cluster | 46 | 97.8947368421 | 263 | 3.4e-70 |
| ACB12552.1 | Fum3 | BGC0000063 | Fumonisin\_biosynthetic\_gene\_cluster | 47 | 98.5964912281 | 262 | 5.9e-70 |
| CBF87240.1 | toxin\_biosynthesis\_proten\_(Fum3),\_putative\_(AFU\_orthologue;\_AFUA\_3G14710) | BGC0000680\_c2 | Austinol/dehydroaustinol,\_locus\_1\_biosynthetic\_gene\_cluster | 39 | 102.807017544 | 203 | 3.2e-52 |
| EAU29533.1 | predicted\_protein | BGC0000682 | Terretonin\_biosynthetic\_gene\_cluster | 35 | 92.2807017544 | 180 | 3.8e-45 |
| ANY57891.1 | PenM | BGC0001372 | Penigequinolone\_biosynthetic\_gene\_cluster | 36 | 91.9298245614 | 166 | 5.7e-41 |
| BAH24000.1 | alpha-ketoglutarate\_dioxygenase | BGC0000356 | Fumitremorgin\_biosynthetic\_gene\_cluster | 36 | 90.8771929825 | 157 | 2.7e-38 |
| AAD32731.1 | MmcH | BGC0000915 | Mitomycin\_biosynthetic\_gene\_cluster | 28 | 56.1403508772 | 71 | 1.9e-12 |
